# What drives diversification of national food supplies? A cross-country analysis

**DOI:** 10.1016/j.gfs.2017.05.005

**Published:** 2017-12

**Authors:** Samira Choudhury, Derek Headey

**Affiliations:** a School of Economics, University of Adelaide, Adelaide, Australia; b Poverty, Health, and Nutrition Division, International Food Policy Research Institute, Washington, DC, USA

**Keywords:** Dietary diversity, Diversification of food supplies, Nutrition, Food systems, Economic transformation

## Abstract

Little previous research has explored what drives the diversification of national food supplies (DFS) across countries and regions. We construct and analyse a cross-country dataset linking a simple DFS indicator - the share of calories supplied by nonstaple foods - with structural transformation and agroecological indicators. Panel econometric models show that several indicators of structural transformation (economic growth, urbanization and demographic change) are strong predictors of diversification within countries, yet time-invariant agroecological factors are also significantly associated with diversification, which appears to explain why some countries have exceptionally low or high DFS relative to their level of economic development. We discuss the implications of these findings for food and nutrition strategies.

## Highlights

• Diversification of food supplies essential for improving nutrition.
• First cross-country study explaining diversification of food supplies (DFS) with panel regressions.
• Structural transformation indicators strongly predict DFS change over time within countries.
• Yet several agroecological factors constrain diversification.
• Countries facing these constraints require special policy attention to improve diets.

## 1 Introduction

The widely used UNICEF framework emphasizes that nutrition outcomes are the product of both food and nonfood factors (UNICEF, 1990). However, for many economists and food security experts “food” in a nutritional context has historically been directly equated to calories, such that countless economic studies analysing the demand for calories (see Bouis et al., 1992 for a review). Nutritionists, in contrast, have increasingly emphasized the importance of a wide range of nutrients. A healthy human diet requires at least 51 known nutrients in consistently adequate amounts (Graham and Tetroe, 2007); moreover, it is likely that there exist many synergies between nutrients, making dietary diversity an important concept in its own right. Nutritionists have therefore developed a wide range of dietary diversity indicators – ranging from detailed weighing of individual food intake to very simple recalls of broadly defined food groups – that have been shown to be strong predictors of adequate nutrient intake (Hatloy et al., 1998, Shimbo et al., 1994, Foote et al., 2004, Steyn et al., 2006, Moursi et al., 2008) and health/nutrition outcomes (Arimond and Ruel, 2004, Kant et al., 1993, Slattery et al., 1997, Levi et al., 1999). Economists, too, are increasingly exploring the merits of such indicators (Headey and Ecker, 2013, Tiwari et al., 2013), and going beyond calories to examine the determinants of household demand for micronutrients (e.g. Ecker and Qaim, 2011).

Despite growing interest in dietary diversity at the individual or household level, there is relatively little research on “food systems” (Herforth and Ahmed, 2015), and very little research on diversification of food *supplies* (hereafter DFS). After many years of focusing solely on calorie supplies, the Food and Agriculture Organization of the United Nations (FAO) recently added a simple DFS measure – the share of calories supplied by non-staple foods – to its expanded suite of food security indicators (FAO, 2016). Headey (2013) shows that this indicator has stronger correlations with maternal and child undernutrition outcomes than the FAO estimates of total calories per capita, despite both indicators being derived from the same set of food balance sheets. Remans et al. (2014) likewise find that this indicator – as well as other DFS indicators – is negatively correlated with national estimates of stunting, wasting, and underweight prevalence (but not overweight prevalence). Thus, as with individual and household measures of the diversity of diets, DFS is negatively associated with indicators of the prevalence of undernutrition and may therefore be a useful metric for monitoring how well a food system supplies a diverse range of nutrients at an aggregate level, which is a *necessary but not sufficient condition for diverse diets among the population as a whole*.

Clearly there are important rationales for measuring and monitoring DFS. However, scarcely any research systematically studies the distribution of DFS across countries, and the evolution of DFS over the course of economic development and structural transformation. In this study we hypothesize that various processes of structural transformation are likely to be strong predictors of DFS. Our conceptualization of structural transformation goes beyond the traditional focus on shifts in the composition of production associated with Chenery and Syrquin (1975), for example, to a more comprehensive definition that incorporates the multiple economic and demographic changes that take place over the course of long run growth and development (Timmer, 2009a). This includes shifts in production and employment out of agriculture, but also increasing commercialization and diversification within agriculture, and a significant shift from a high fertility and predominantly agrarian demographic profile to a low fertility and more urbanized demographic profile. But while historical evidence suggests the DFS does increase over the course of development, microeconomic theories of imperfect markets suggests that the speed of diversification may vary substantially. In particular, the tradability of many highly perishable non-staple foods limited also means that deeper structural factors in the agricultural sector may also explain long run differences in DFS, even between countries at similar income levels.

Various types of studies have investigated the role of income in influencing dietary diversification. Theil and Finke (1983), using cross-sectional data for 30 countries, document that the diversity of food supplies increases as income per capita rises. Behrman and Deolalikar (1989) use survey data from India to document strong household preferences towards greater variety of food as incomes increase, but Subramanian and Deaton (1996) find that demand for diverse foods only tends to increase once demand for more calories has satiation. Using cross-country data from the International Comparison Program (ICP), Seale et al. (2003) and Muhammad et al. (2011) confirm that staple foods indeed have smaller income elasticities compared to nonstaple foods such as meat and dairy, and those differences tend to rise with income. Melo et al. (2015) conduct a meta-analysis of food-income elasticities in sub-Saharan Africa, and find much higher elasticities for animal sourced foods.

More systems-oriented research on structural transformation also describes changes in food systems and concomitant changes in diets. The development of larger urban markets with high concentrations of wealthier consumers fosters more sophisticated value chains for nutrient-rich foods (Reardon and Timmer, 2012). The emergence of these value chains can have transformative feedback effects on the agricultural sector, especially those with good access to urban centers, which allows them to concentrate production into higher value products. Structural transformation also entails demographic changes in the form of declining fertility rates and age dependency ratios, and increased educational attainment. As the proportion of children in a given population declines, disposable incomes tend to increase (Bloom and Williamson, 1998), though reduced age dependency ratios may also encourage parents to devote more resources to fewer children (Becker and Lewis, 1973). Indeed, micro-level demand analyses also regularly incorporate basic demographic indicators. And independent of any effects on fertility rates, education may increase the demand for nutrient-rich foods because of improved nutritional knowledge (Alderman and Headey, 2014, Block, 2004, Webb and Block, 2004).

In this paper we explore associations between the diversification of food supplies and a range of structural economic, demographic and infrastructural and agroecological factors with the aid of a rich cross-country dataset (described in Section 2). We first document some basic stylized facts regarding the distribution of DFS across countries, and the evolution of DFS over time and over the course of economic growth (Section 3). Consistent with expectations, DFS is strongly associated with economic growth and other structural transformation indicators, although some countries have unusually high or unusually low levels of DFS even after controlling for levels of economic development. We then use these data to conduct more rigorous tests of these hypotheses using fixed effects models (which only test the effect of time-varying structural transformation factors) and correlated random effects models (which also permit the inclusion of time-invariant structural factors, such as agroecological characteristics). We find that economic growth, urbanization and demographic change successfully explain DFS changes over time, but also that time-invariant structural factors (for example, land constraints) substantially explain the persistence of DFS differences across countries at similar levels of development (Section 4). Moreover, these results are robust to alternative indicator of DFS – the share of calories sourced from animal foods – and to the inclusion of alternative explanatory variables (Section 5). Our concluding remarks discuss the implications of this research for food and nutrition strategies in particular (Section 6). Our principal conclusion is that while structural transformation can indeed be expected to bring about the diversification of food supplies, some countries face greater structural barriers to this diversification process because of the particular characteristics of their agricultural production systems and the limited tradability of nutrient-rich nonstaple foods. These countries require more aggress strategies to accelerate dietary diversification through both supply and demand-side interventions. How best to do so should be the subject of future research.

## 2 Data and methods

### 2.1 Data

In this paper we use a cross-country panel dataset that merges data from a variety of sources. Food balance sheets from the (FAO) containing data on the supply of calories, proteins and fats from different food groups (for example, cereals, vegetables) have been used to create a very simple measure of diversity of food supply which is our outcome variable: calories supplied from nonstaple foods, where staple foods include cereals and root crops. These data have well known weaknesses. Conceptually, they do not measure the composition of diets per se, but only food supply in aggregate, and much food may unequally distributed within a population. Empirically, the underlying quality of national data sources that go into FAOSTAT is often poor in low-income countries. Nevertheless these data remain the only comprehensive means of comparing the calorie content and diversity of food supplies across countries and over time. Moreover, while these data may be weakly associated with measured adult intake of particular foods (Del Gobbo et al., 2015), Fig. A1 in the Appendix shows that DFS is strongly correlated with a child level indicator of dietary diversity, the share of children 6–24 months consuming a “minimal acceptable diet” of 4 or more food groups.

We use a wide range of indicators to explain DFS patterns and trends. We use private consumption expenditure per capita (measured in international purchasing power parity [PPP] dollars from the World Bank (2016) as the most suitable indicator of household purchasing power (since GDP per capita includes government expenditures that may be less relevant for influencing food demand); average years of schooling attained, measured at five-year intervals, as our indicator for education (Barro and Lee, 2010); and the share of urban population and the share of children in the population aged 0–14 years as indicators of urbanization and the demographic transition (World Bank, 2016). The World Bank (2009) also reports data on measures of topography (hills and mountains, lowland areas) that might influence production diversity. For example, hill areas are suitable for orchards, whilst wet lowland areas tend to be less suitable for vegetable production. Likewise rural population density can be considered a proxy for land constraints. Smaller farms may encourage diversification into higher value crops (Boserup, 1965) or small farms may proxy for rural poverty, which may constrain demand for more nutrient-rich foods. For infrastructure indicators, we include road density, international shipping costs, and electric power consumption to this sample. Though time-varying in principle, these indicators are only available for fixed points in time. Another set of time invariant geographical characteristics (from WorldClim – Global Climate database) contains data on average monthly rainfall and standard deviation. Cross-country average groundwater values have also been derived from a global groundwater database (Fan et al., 2013). Water availability could have complex relationships with production diversity. More rainfall, and more stability in rainfall, might reduce risk and encourage poor farmers to diversify production. On the other hand, too much water – especially groundwater – can lead to waterlogged soils that are poorly suited for vegetable production, leaving rice, in particular, as the only option for water-abundant lowland systems.

After integrating the datasets, we have a five- year unbalanced panel data set of 51 countries (low to high-income countries) for the period 1965–2010, with a sample of 557 observations, although the actual sample size used varies according to the model specification ( Table A3 in the appendix lists the countries). Table 2.1 reports descriptive statistics. The sample covers ample variation in DFS, and in the main indicators of structural transformation, although we also test robustness to sample restrictions.

**Table 2.1:** Descriptive statistics for key variables.

Variable	Observations	Mean	Standard deviation	Min	Max
Share of calories supplied from nonstaples (0–1)	557	0.49	0.16	0.14	0.78
Share of proteins from animal-sourced foods (0–1)	557	0.40	0.17	0.09	0.70
Consumption expenditure (constant PPP $)	557	5753	5906	144	27,044
Education (years)	557	5.92	2.83	0.13	12.03
Urban population (% of total population)	557	51.59	20.96	3.58	92.49
Population ages 0–14 (% of total population)	557	34.11	10.32	13.29	49.97
Electric power consumption (kWh per capita)	557	27.20	25.40	0.03	99.32
Road density (roads per 1000 sq. km)	557	74.59	90.72	3.60	372.20
Shipping costs (global rank)	557	70.31	46.68	2.00	169.00
Suitable land (%)	557	61.92	21.82	20.64	97.63
Population density (per 1000 m^2^)	557	111.82	164.52	0.39	1164.14
Hills and mountains (% of total land area)	557	0.52	0.30	0.00	1.00
Lowlands (% of total land area)	557	0.25	0.23	0.00	1.00
Groundwater (meters)	557	1.91	1.19	0.07	4.82
Average rainfall (mm)	557	90.85	49.53	27.60	227.00
Rainfall variation (mm)	557	51.39	39.95	6.40	169.50

Note: PPP = international purchasing power parity; kWh = kilowatt-hour.

## 3 Methods

In terms of methods, we implement a variety of statistical techniques, via Stata version 14.0, to analyse these data. Graphically, we apply nonparametric techniques, specifically the local polynomial smoother with 95 percent confidence intervals (the *lpolyci* command) to look for any nonlinear relationships in key parameters of interest. To econometrically explore factors that might influence DFS, we first estimate fixed-effects (FE) models to assess the associations between diversity of food supply *(DFS)* and four time-varying intermediate determinants (consumption, education, urbanization and population 0–14 years), with trend effects represented by a vector of year dummy variables *(T).* These regressions are effectively difference-in-difference regressions, though a disadvantage of fixed effects models is that researchers are sometimes directly interested in the impacts of time-invariant factors. We therefore also utilize the correlated random effects (CRE) model, also called the Chamberlain-Mundlak model, following Mundlak (1978) and Chamberlain (1984), to account for the panel structure in the data whilst still allowing coefficients of time-invariant independent variables to be identified. In this model fixed effects are effectively replaced with country averages of time-varying indicators as well as a vector of time-invariant indicators of interest (for example, agroecological indicators). This model still therefore still specifies within-country effects of time-varying indicators, but allows us to test associations between time-invariant factors and DFS. The key assumption is that the remaining unobserved heterogeneity is uncorrelated with the independent variables.

## 4 Descriptive results

**Fig. 3.1:**
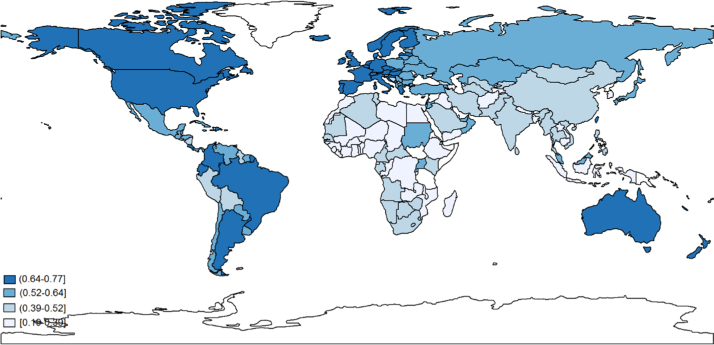
calories supply from nonstaples across countries, 2010. Notes: Each country is colored according to value of corresponding diversity of food supply.

Source: Authors’ estimates from FAO (2016) data.

How does DFS vary across regions and income levels? In this section we assess patterns and trends in DFS to understand some basic stylized facts. Fig. 3.1 presents a map of the DFS across countries for 2010, with darker shades of blue representing greater DFS. Unsurprisingly, the map indicates that North America, Western Europe, Australia, New Zealand and several Latin American countries show the highest levels of diversity of food supply, followed by Eastern Europe, Japan, several Latin American countries and Japan. In contrast, South Asian countries have low DFS, particularly Bangladesh and Nepal. In Africa there is some heterogeneity, but DFS is very low in West Africa, Ethiopia and much of southern Africa.

**Fig. 3.2:**
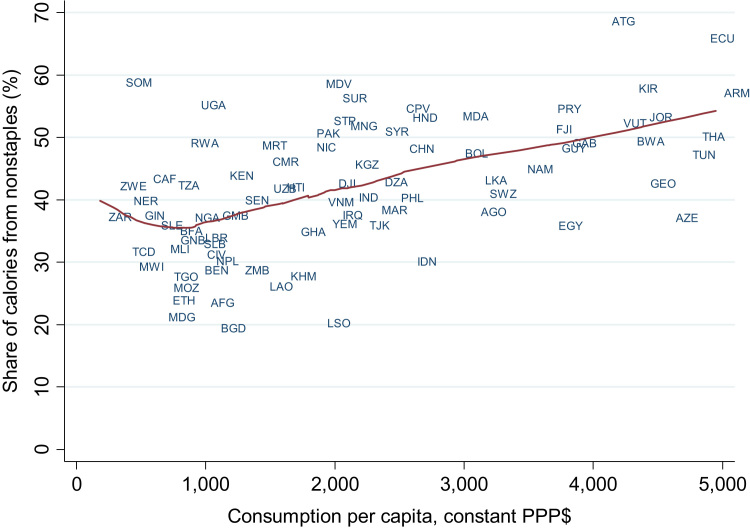
LOWESS and scatter plots of diversity of food supply (% of calories from non-staples) against household consumption per capita for low and low middle income countries, 2010.

Source: Diversity of food supply is sourced from FAO (2016), and consumption per capita is sourced from the World Bank (2016). Note: The solid line is an lpoly plot estimated in Stata 14. LOWESS = locally weighted scatterplot smoothing; PPP = purchasing power parity. Three-letter World Bank country codes denote specific observations. The full list of corresponding country codes can be found at: http://wits.worldbank.org/WITS/wits/WITSHELP/Content/Codes/Country_Codes.htm.

Fig. 3.2 plots DFS against consumption per capita for 2010, with a LOWESS line showing the predicted relationship. Consumption per capita is certainly strongly associated with DFS although there is substantial variation around the predicted relationship. For example, several large countries have unusually low DFS, notably Bangladesh (BGD), Indonesia (IDN) and Egypt (EGY), while several other rice consuming countries also lie below the prediction line, such as Madagascar (MDG), Laos (LAO) and Cambodia (KHM).

Table 3.1 reports trends in DFS for income groups, major regions and countries over the1961-2010 period. For income groups (as defined by 2010 data), the major result is that dietary diversification was slow in the poorest income groups, with just a 5 percentage point increase over the time period for both the low income and lower middle income groups. In contrast, upper middle income and high-income Organization for Economic Cooperation and Development (OECD) groups saw 11-point and 9-point changes over this period. Regionally, Latin America and the Caribbean saw moderate changes on average, though changes in countries like Mexico and Brazil were rapid (14 point changes in both countries). Africa south of the Sahara has seen very little diversification in food supplies, just 5 points on average, and there is relatively little variation in DFS changes. In South Asia Pakistan saw a sizable improvement in DFS (11 points) but changes in India and Bangladesh were very modest (3 and 4 points). In East Asia and the Pacific, China and Vietnam experienced dramatic increases in DFS (25 and 23 points respectively), but Indonesia saw only a modest 6-point increase.

**Table 3.1:** Trends in calories supply from nonstaples for income groups, major regions and countries, 1961–2010.

	**Sample median: 1961**	**Sample median:2010**	**Change in median**
**High income OECD**	61%	70%	9%
Japan	34%	58%	24%
**Upper middle income**	44%	55%	11%
**Lower middle income**	35%	40%	5%
**Lower income**	25%	30%	5%
**Latin America & Caribbean**	48%	55%	7%
Mexico	41%	55%	14%
Brazil	50%	65%	14%
**Sub-Saharan Africa**	32%	36%	5%
Nigeria	34%	34%	0%
Kenya	34%	41%	7%
**South Asia**	30%	37%	7%
India	36%	39%	3%
Pakistan	40%	51%	11%
Bangladesh	15%	19%	4%
**East Asia and Pacific**	33%	43%	10%
Vietnam	16%	39%	23%
China	23%	48%	25%
Indonesia	23%	29%	6%

Note: OECD = Organization for Economic Cooperation and Development; SSA = Africa south of the Sahara.

**Fig. 3.3:**
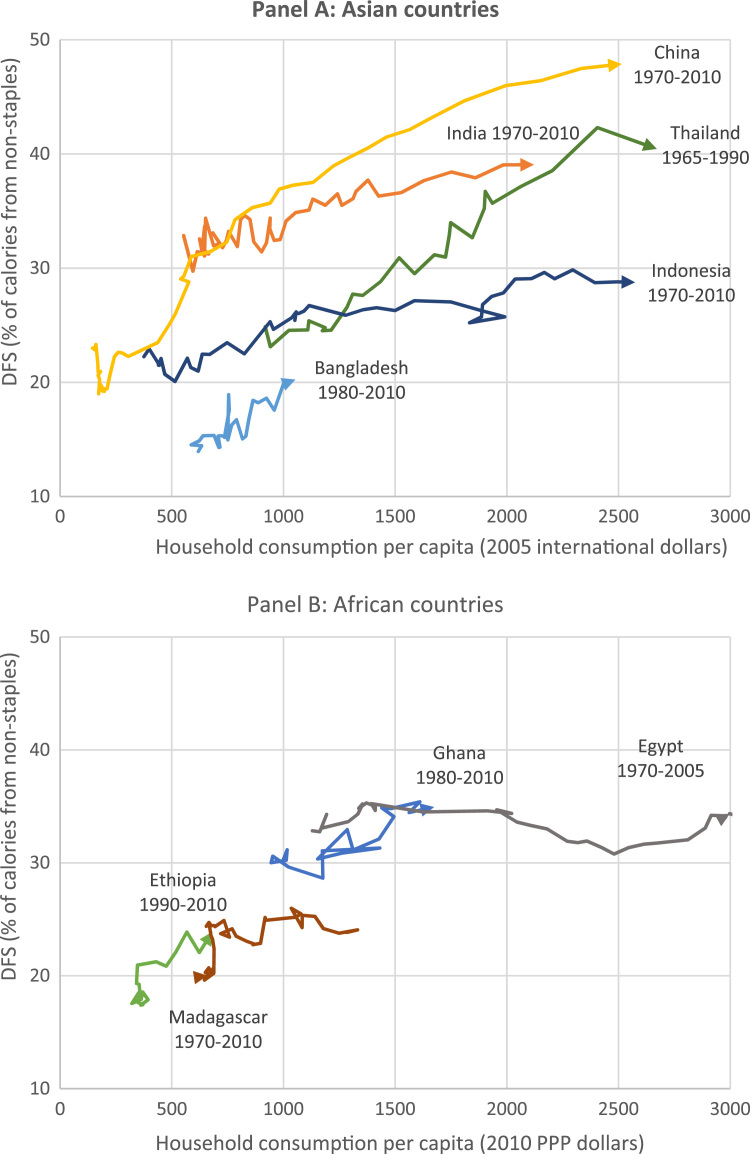
Long run relationships between DFS and consumption per capita over time for selected countries. Note: PPP = purchasing power parity.

Source: Authors’ estimates from World Bank (2016) and FAO (2016) data sources.

Clearly one explanation of the patterns in Table 3.1 is the variation in income growth across counties. Fig. 3.3 therefore plots DFS trajectories against changes in consumption per capita. Panel A focuses on Asian countries and Panel B on African countries. In Panel A, China offers a remarkable example of extremely rapid diversification of food supplies. In the 1970s nonstaple foods accounted for just 20 percent of China's calorie supply, but as economic growth accelerated from 1978 onward that ratio rose to almost 50 percent. Thailand has followed a somewhat similar trajectory. Like China and Thailand, Indonesia had similar dependence on rice and other staples in the 1960s and 1970s, but Indonesia's food basket appears to have diversified slowly, with nonstaples accounting for just 30 percent of the total supply of calories by 2010. Moreover, this slower diversification is only partly explained by lower rates of economic growth: Indonesia's food supply in 2010 was much less diversified than Thailand's was in 1990, when their income levels were comparable. India appears to follow a more intermediate trajectory. Diversification during the 1970s and 1980s (the heyday of India's Green Revolution in wheat and rice) was very modest, but it picked up during the 1990s and 2000s during a period of more rapid economic growth. Bangladesh has experienced more modest but solid growth in consumption since the mid 1990s, and its food supply has begun to diversify from its extremely low base. However, its food supply in 2010—when annual consumption averaged about $1000 per capita—is about half as diversified as China's food supply at a comparable level of consumption (19 percent versus 37 percent).

In Panel B we look at African countries. Egypt has followed a flat DFS trajectory, with scarcely any diversification of food supplies over the course of reasonably rapid and prolonged growth in per capita consumption. Ethiopia has even lower levels of consumption but recent economic growth also appears to be translating into a more diverse food supply. Ghana follows a similar pattern, albeit from a more diversified starting point. Finally, Madagascar is a contrary example of a country where per capita consumption has actually fallen over time, from around $1300 per capita in 1970 to just $700 per capita in 2010.

Overall, DFS appears to be very strongly associated with levels of development in general, and per capita consumption levels and growth rates. That said, there are striking deviations from this expected relationship that are likely related to factors not captured by average income. Most notably, DFS is exceptionally low in several countries characterized by high rates of rice production and irrigation. In such countries growth in per capita consumption still seems to be driving diversification of food supplies, but from a much less diversified base (for example, Bangladesh) and in some cases at a much slower rate (for example, Indonesia, Egypt).

## 5 Regression results

Table 4.1 reports FE and CRE regressions of diversity of food supply against time-varying indicators of structural transformation, as well as a series of time-invariant indicators of infrastructure and agroecological characteristics. The regressions are semi-log, meaning that all coefficients reflect the change in the share of calories supplied from nonstaples resulting from a 100 percent increase in the explanatory variable. Hence the coefficients of the different indictors are highly comparable. We also note that both the FE and CRE models produce high coefficients of determination, suggesting that these specifications do a good job in predicting spatial and temporal variation in DFS.

**Table 4.1:** Correlated Random Effects and Fixed Effects regressions of the semi-log DFS model.

Estimator	FE	CRE
*Time-varying indicators*		
Consumption per capita	0.055***	0.059***
	(0.006)	(0.012)
Education (years)	0.006	0.014
	(0.012)	(0.024)
Urban population	0.066***	0.067***
	(0.011)	(0.023)
Population ages 0–14 years	−0.095***	−0.088**
	(0.018)	(0.036)
*Time-invariant indicators*		
Electricity consumption		−0.003
		(0.012)
Road density		0.039***
		(0.004)
Shipping costs		−0.002
		(0.004)
Suitable land		0.024***
		(0.007)
Population density		−0.114***
		(0.012)
Hills and mountains		0.006**
		(0.003)
Lowland areas		0.002
		(0.003)
Groundwater depth		−0.014***
		(0.005)
Average rainfall		−0.028***
		(0.010)
Rainfall variation		−0.011
		(0.007)
Time effects	Yes	Yes
R-squared		0.870
R-squared within	0.624	
Number of observations	557	557

Standard errors are in parentheses. All explanatory variables are in logs.

*p<0.10.

** *p*<0.05.

*** *p*<0.01.

As the nonparametric results suggested, per capita consumption expenditure is a strong predictor of changes in diversity of food supply, even in terms of within-country effects. For every doubling of household consumption expenditure, calories supplied from nonstaples will go up by nearly 6 percent points, an association that is strongly significant at the 1 percent level in both FE and CRE regressions. However, other indicators of structural transformation are also highly significant. Indeed, point estimates of the partial elasticity of DFS with respect to urbanization are somewhat larger than that of consumption per capita (though not significantly so). Even more strikingly, the population aged 0–14 years has a large, negative, and highly significant association with DFS in both the FE and CRE models. Moreover, these associations are significantly larger than the coefficients on consumption per capita. Perhaps surprisingly, however, we do not find any significant association between the years-of-education variable and DFS.

As noted in Section 2, the CRE model also allows us to test associations between DFS and time-invariant factors. In terms of transport infrastructure, the partial elasticity for road density is 0.04, while the elasticity associated with shipping costs is insignificant. This likely reflects the fact that many nutrient-rich foods are highly perishable and are not shipped large distances.

Consistent with economic theories of highly imperfect markets in underdeveloped rural settings, we observe some significant associations between DFS and various agroecological characteristics. Land suitability for crop production is positively and significantly associated with DFS, though the estimated partial elasticity is modest in magnitude (0.02). More strikingly, rural population density has a relatively large and negative partial elasticity of −0.11. We interpret rural population density as a proxy for land constraints, which may operate through several channels, such as feed constraints that inhibit production of animal-sourced foods, and a greater prevalence of small farms that push farmers into intensive cereal cultivation. Lowland areas have an insignificant association, but hilly and mountainous countries appear to have somewhat higher DFS. Groundwater depth has a negative association with DFS, but rainfall is also negatively associated with DFS.

Finally, both models produce large coefficients of determination, suggesting that these specifications do a good job in predicting spatial and temporal variation in DFS. In the FE model the within R-squared is 0.62, suggesting that these four structural transformation indicators explain around two-thirds of the changes in DFS over time. The aggregate R-squared in the CRE model factors in the explanatory power of both time-varying and time-invariant factors, but it is worth noting that the time-invariant factors explain a high share of the total variation in DFS (56 percent). Overall, the results support Bennett's (1941) prediction that economic growth leads to diversification of food supplies, but the models are also highly consistent with broader theories of structural transformation (Section 1).

We now use these regression results to analyse the predicted sources of DFS change over time using a simple decomposition at means technique, the results of which we report in Table 4.2.^1^ These decompositions are based on the fixed-effects regressions reported above, since the time-invariant indicators in the CRE model obviously cannot explain changes over time. The first column reports the estimated coefficient from that regression. The next three columns respectively report the 1961 and 2010 sample means and the change in means across time. The last column reports the share of predicted change accounted for by each variable. To see how these figures are derived, consider the second row of column 5 of Table 4.2, which reports the predicted change in DFS, which is the mean change in consumption per capita from 1961 to 2010 multiplied by the coefficient of consumption per capita on DFS from regression 1 in Table 4.1. This calculation suggests that increases in consumption per capita from 1961 to 2010 resulted in a 0.06 percent point increase in the share of calories supplied from nonstaples. In other words, among the sources of predicted change, consumption per capita stands out as the single largest factor, explaining 41 percent of the predicted change in diversity of food supply. We also observe sizable contributions from urbanization (0.04) and reductions in the share of the population aged 0–14 (0.04). In aggregate the model predicts an average change in DFS of 13 percent points, which is slightly more than the actual change observed (11 points). Overall, though, it appears that these three structural transformation indicators do a good job of predicting changes in DFS over time.

**Table 4.2:** Decomposing sources of DFS change for the full sample, 1961–2010.

	Estimatedβ	Sample mean: 1961	Sample mean: 2010	Change in mean	Predicted DFS change	Share of predicted DFS change
Diversity of food supply		0.43	0.52	0.10	0.13	100%
Consumption per capita	0.06	7.58	8.58	1.00	0.06	41%
Urban population	0.07	3.47	4.09	0.62	0.04	31%
Population ages 0–14	−0.10	3.63	3.23	−0.40	0.04	28%

## 6 Robustness tests

In this section we engage in a series of robustness checks designed to establish the extent to which the results presented in the previous section stand up to alternative specifications and to a more confident causal interpretation.

Table 5.1 reports FE and CRE regression results using an alternative indicator of DFS: the share of proteins supplied by animal-sourced foods (ASF supply). While dietary diversity in general may be beneficial for nutrition, animal-sourced foods have been identified as particularly important for child growth outcomes and of course for reducing deficiencies in key micronutrients (Hoppe et al., 2006, Iannotti et al., 2013; Murphy and Allen, 2003). Overall the pattern of coefficients in Table 5.1 is very similar to that of Table 4.1, though there are some differences as well. The coefficients on consumption are somewhat larger in the context of ASF supply. One difference in the animal-sourced food results is that the coefficient on urbanization is insignificant in all the regressions reported in Table 5.1, but the share of the population aged 0–14 years still yields a large and negative elasticity. The CRE results reveal positive but modest associations between ASF supply and electricity supply and road density, but there is again no association with shipping costs. As with the result in regression 2, land suitability is positively associated with ASF supply but population density is negatively associated, which may reflect lack of feed or other sources of comparative disadvantage in producing animal-sourced foods. Groundwater depth is again negatively associated with the dependent variable, but rainfall has a positive association with ASF supply, perhaps indicating the importance of rainfall for increasing feed availability.

**Table 5.1:** Correlated Random Effects and Fixed Effects regressions of the semi-log DFS model using alternative indicator of diversity of food supply: Protein share of animal-sourced foods.

Estimator	FE	CRE
*Time-varying indicators*		
Consumption per capita	0.063***	0.059***
	(0.008)	(0.012)
Education (years)	0.012	0.011
	(0.015)	(0.024)
Urban population share	0.023	0.030
	(0.014)	(0.023)
Population aged 0–14 years	−0.155***	−0.017***
	(0.023)	(0.037)
*Time-invariant indicators*		
Electricity consumption		−0.004
		(0.012)
Road density		0.012**
		(0.004)
Shipping costs		−0.001
		(0.004)
Suitable land		0.031***
		(0.007)
Population density		−0.128***
		(0.011)
Hills and mountains		−0.006*
		(0.003)
Lowland areas		0.006
		(0.003)
Groundwater depth		−0.034***
		(0.005)
Average rainfall		0.040***
		(0.010)
Rainfall variation		−0.010
		(0.007)
Time effects	Yes	Yes
R-squared		0.885
R-squared within	0.541	
^Number of observations^	557	557

Standard errors are in parentheses. All explanatory variables are in logs.

* *p*<0.10.

** *p*<0.05.

*** *p*<0.01.

Next we estimate CRE and FE models where diversification of production as the dependent variable rather than diversification of total food supply. The difference between the two indicators stems from net imports, but Remans et al. (2014) show that trade is a less important source of DFS in lower income countries, so one might expect the DFS results reported above to be reasonably robust to using production diversity as a dependent variable. Table A3 in the Appendix reports the results of this change. The elasticities of consumption per capita are indeed very similar to the DFS results reported above, as is the elasticity with respect to the population share of persons 0–14 years of age. However, the elasticity on urbanization is not significant, suggesting that urbanization is primarily associated with increased reliance on food trade. Strikingly, the infrastructural and agroecological coefficients remain very similar, suggesting that these factors do indeed influence DFS by conditioning what can and cannot be domestically produced.

Next, we consider a series of different indicators of agricultural and trade policies—tariff rate, agricultural tax and subsidy, price level of consumption, and public spending on agriculture. These indicators were not available for all countries and all years, and were therefore omitted from the results reported in the previous section. Another limitation is that these indicators is that yield little information on diversification specifically. For example, substantial proportions of government expenditures in developing countries are thought to be targeted towards staple grains. Grains-oriented policies could still help or hinder diversification, depending in part on whether they reduce the relative price of grains. A third limitation is that international policies – such as investment in international agricultural research – are also critically important, but very difficult to measure at a national level. Even so, we believe there is merit in exploring whether agricultural policy indicators bear any association with GFS.

Table A4 in the Appendix reports FE results from including these indicators. In regression 1 we include indicators of real rates of assistance to agriculture (RRA) from the World Bank agricultural distortions database (Anderson, 2008). We bifurcate the RRA indicators into subsidies and taxes to allow for asymmetric effects. Positive RRA values are agricultural subsidies and negative RRA values are agricultural taxes. We find a positive and significant partial elasticity of DFS with respect to agricultural subsidies, though the association is small in magnitude (0.02). We find no significant effects of agricultural taxation. For a smaller subset of countries we also tested whether subsidization of staple foods had any association with DFS, but we found no evidence that it did (results available on request). However, the Anderson (2008) study found that in developing countries relatively few fruits, vegetables, and animal-sourced foods were internationally tradable, and hence RRA values for those food groups are missing for many developing countries.

We find no effect of agricultural tariff rates on DFS, and do not find any association between the price level of consumption (the PPP for consumption relative to the official exchange rate) and DFS, which might capture implicit taxation of tradable goods and services. However, we do find a small positive association between public spending on agriculture and DFS. Thus there is some evidence that greater public support for agriculture stimulates diversification, although the associations are modest and likely to be heterogeneous.

## 7 Conclusions

In this paper we set out to systematically explore what drives the diversification of food supplies across countries and regions, and over the course of economic development. We first show that a range of economic theories and evidence predict that food systems should diversify throughout the course of economic growth. Existing economic evidence on DFS and diets is largely confined to the indirect evidence provided by food demand analysis. Income elasticities estimated from such microeconomic studies suggest that diversification into animal-sourced foods and processed foods is more rapid than diversification into fruits, vegetables, and other crop-based foods. However, microeconomic theories of market failures in underdeveloped settings also predict that agroecological factors condition DFS given the high degree of perishability of many nutrient-rich nonstaple foods, and therefore the limited scope for trade in such foods.

Both our descriptive evidence and our more formal regression models are consistent with these theories. We find strong support for Bennett's (1941) law—DFS is strongly associated with economic growth—but also evidence that other forms of economic transformation drive DFS, notably urbanization and the demographic transition from younger to older populations. This last association is particularly strong. We hypothesize that the transition to an older population structure may influence disposable income at any given per capita level of income, though it may also shift preferences toward tastier and more nutrient-rich foods. The association between DFS and urbanization is also but seemingly driven by linkages between urbanization and increased reliance on food trade.

Yet although economic transformation is clearly a very important driver of diversification, evidence herein suggests that some countries have unusually undiversified food supplies relative to their development levels. Descriptively, we note that many countries that are major consumers and producers of rice seem to have undiversified food supplies—examples being Bangladesh, Indonesia, Madagascar, Cambodia, and Laos. One explanation may be statistical in nature, particularly if stocks of rice are relatively large in some countries or simply overestimated (for a discussion on the difficulties of estimating rice stocks, see Timmer, 2009b). But another explanation may be that these countries share agroecological characteristics that give them a comparative advantage in rice production, and a comparative disadvantage in the production of noncereal foods. Our regression analysis provides some support for this hypothesis, with high levels of population density being strongly negatively associated with DFS. High population density may be associated with lack of feed for the production of animal-sourced foods—results from our robustness tests support that hypothesis—although high population density may also be associated with abundance of water via either irrigation or rainfall, and there is some evidence that waterlogged soils are a constraint to diversification into fruit and vegetable production. High population density is also a proxy for land availability, however, and it may be that land constraints somehow inhibit diversification out of staples.

The analysis is this paper is subject to important limitations. It is well known that the FAO food balance sheets provide estimates that have considerable errors, some of which may be systematic in nature. Del Gobbo et al. (2015) compare FAO measures of food supply per capita with household survey–based estimates and find that the FAO measures tend to underpredict consumption of most food groups. A second limitation is that our study does not have an experimental design. Rather we focus on testing whether conditional relationships in the data are consistent with economic theory. However, the use of fixed-effects and correlated-random-effects models at least strengthens the rigor of these tests, and rules out obvious sources of confounding.

While these inherent limitations in the quality of the underlying data and in the analytical methods used should not be ignored, it is worth reiterating that the regression analysis provides a series of results that are highly consistent with existing economic theory and evidence. Structural transformation is clearly a fundamental driver of the diversification of food supplies, which may provide one explanation as to why measures of economic growth and urbanization have been robustly associated with lower stunting rates in a wide range of studies (see Bershteyn et al., 2015 for a review).

At the same time, this finding poses many challenges for nutrition strategies, policies, and program design, because it illustrates the difficulties of diversifying food supplies and diets in the absence of prolonged economic growth and transformation. Moreover, while there are many nutrition programs that aspire to accelerate dietary diversification, it is still unclear whether such programs can substantially and sustainably improve diets without prolonged growth in incomes (Ruel and Alderman, 2013, Pinstrup-Andersen, 2013). In addition to behavioral change communications strategies that aim to shift household preferences toward more nutritious foods, an additional strategy would involve using food policies to reduce the real price of nutrient-rich foods. To date, however, little research has assessed how countries might best pursue a strategy of making nutrient-rich foods both more desirable and more affordable, and what impact nutrition-sensitive food policies of that nature might have on diets and various nutrition outcomes. This would appear to be an important agenda for future research.

## Footnotes

1 A more flexible alternative to a simple decomposition at means is a Oaxaca–Blinder decomposition, which allows coefficients to vary over time. However, the relatively small size of our sample produces some instability in the size of the coefficients when we restrict regressions to a single cross-section.
