# Supplementary material for: What drives diversification of national food supplies? A cross-country analysis
Source: Glob Food Sec. 2017 Dec;15:85–93. doi: 10.1016/j.gfs.2017.05.005 (PMC5727671; doi:10.1016/j.gfs.2017.05.005)
Supplement: Supplementary file 1 — Supplementary material [file mmc1.docx]

**Appendix: Supplementary Table**

**Table A1. Definitions of variables**

| Variable | Definition |
| --- | --- |
| *Diversity of food supply* |  |
| Calories supplied from nonstaples | The share of calories not derived from starchy staples: cereals, roots, |
|  | tubers, and plantains |
| Proteins from animal-sourced foods | Share of proteins supplied by any food of animal origin |
| Calories produced from non-staples | The share of calories produced that are not derived from starchy staples: cereals, roots, tubers and plantains |
| *Transformation indicators* |  |
| Consumption per capita | Household final consumption expenditure per capita is the market |
|  | value of all goods and services, purchased by households |
| Education (years) | Average years of schooling attained in five-year intervals |
| Urban population (% of total population) | Share of people living in urban areas |
| Population ages 0–14 (% of total) | Share of children in the population aged 0–14 years |
| *Time-invariant geographical and infrastructural characteristics* | |
| Rural population density | The population estimated to be rural divided by agricultural land |
| Electricity consumption (kWh per capita) | The production of power plants less power plant use, transmission, distribution, and transformation losses, divided by midyear population |
| Road density (%) | Total length of the road network—includes the length of the paved and unpaved portions divided by country land area |
| Shipping costs | Country ranking of international shipping costs |
| Groundwater depth (meters) | Average groundwater values in total land area |
| Hills and mountains (% of total land area) | The share of hill and mountain areas |
| Lowlands (% of total land area) | The share of lowland areas in total land area |
| Suitable land (%) | The share of land suitable in total land area |
| Rainfall (mm) | Average monthly rainfall, 1980–2000 |
| Rainfall_std (mm) | Standard deviation of monthly rainfall over 1980–2000 |

Source: FAO (2016); World Bank (2009, 2016); Barro and Lee (2010); WorldClim (2016).

Note: kWh = kilowatt-hours.

**Table A2. Fixed Effects regressions of the semi-log DFS model using an alternative indicator of education**

| Estimator | FE | FE |
| --- | --- | --- |
| Dependent variable | Calories from nonstaples | Calories from nonstaples |
| Education (years) (Barro and Lee) | 0.006 |  |
|  | (0.012) |  |
| Education (years) (Cohen and Soto) |  | 0.028 |
|  |  | (0.018) |
| Consumption per capita | 0.055*** | 0.051*** |
|  | (0.006) | (0.009) |
| Urban population | 0.066*** | 0.061*** |
|  | (0.011) | (0.014) |
| Population 0–14 years | -0.095*** | -0.119*** |
|  | (0.018) | (0.025) |
|  |  |  |
| Time effects | Yes | Yes |
| R-squared within | 0.624 | 0.662 |
| Number of observations | 557 | 304 |

Source: Authors’ estimates.

* *p* < 0.10. ** *p* < 0.05. *** *p* < 0.01. Standard errors are in parentheses.

Table A3 reports results from adding alternative indicators of education. The education indicator used in the previous section was years of schooling from Barro and Lee (2010) but (Cohen and Soto, 2007) criticized the quality of that data and produced an alternative series measured every 10 years. The coefficient on years of education is still insignificant, however, and there is no evidence that education contributions to the diversification of food supplies above and beyond any impact it has on the other three structural transformation processes.

**Table A3. Correlated Random Effects and Fixed Effects regressions of the semi-log Diversification**

**of Production model**

| Estimator | FE | CRE |
| --- | --- | --- |
|  |  |  |
| *Time-varying indicators* |  |  |
| Consumption per capita | 0.037*** | 0.062** |
|  | (0.012) | (0.026) |
| Education (years) | -0.004 | 0.059 |
|  | (0.022) | (0.050) |
| Urban population | 0.123*** | 0.097** |
|  | (0.021) | (0.048) |
| Population ages 0-14 years | -0.065* | -0.009 |
|  | (0.034) | (0.076) |
| *Time-invariant indicators* |  |  |
| Electricity consumption |  | 0.032 |
|  |  | (0.025) |
| Road density |  | 0.017** |
|  |  | (0.009) |
| Shipping costs |  | 0.017** |
|  |  | (0.008) |
| Suitable land |  | -0.053*** |
|  |  | (0.014) |
| Population density |  | 0.135*** |
|  |  | (0.024) |
| Hills and mountains |  | 0.026*** |
|  |  | (0.006) |
| Lowland areas |  | 0.004 |
|  |  | (0.007) |
| Groundwater depth |  | 0.036*** |
|  |  | (0.010) |
| Average rainfall |  | 0.014 |
|  |  | (0.021) |
| Rainfall variation |  | -0.011 |
|  |  | (0.015) |
| Time effects | Yes | Yes |
| R-squared |  | 0.505 |
| R-squared within | 0.236 |  |
| Number of observations | 546 | 546 |

Source: Authors’ estimates.

* *p* < 0.10. ** *p* < 0.05. *** *p* < 0.01. Standard errors are in parentheses. All explanatory variables are in logs.

**Table A4. Fixed Effects regressions of the semi-log DFS model using alternative indicators of agricultural and trade policies**

| Estimator | FE | FE | FE | FE |
| --- | --- | --- | --- | --- |
| Consumption per capita | 0.053*** | 0.041* | 0.059*** | 0.049*** |
|  | (0.010) | (0.017) | (0.006) | (0.011) |
| Education (years) | 0.002 | 0.077** | 0.006 | 0.055** |
|  | (0.017) | (0.028) | (0.012) | (0.019) |
| Urban population | 0.096*** | 0.087* | 0.068*** | 0.045* |
|  | (0.016) | (0.039) | (0.011) | (0.019) |
| Population ages 0-14 years | -0.114*** | -0.099** | -0.091*** | -0.090*** |
|  | (0.027) | (0.030) | (0.018) | (0.024) |
| Tariff rate |  | 0.006 |  |  |
|  |  | (0.005) |  |  |
| Agricultural tax | -0.018 |  |  |  |
|  | (0.015) |  |  |  |
| Agricultural subsidy | 0.020* |  |  |  |
|  | (0.009) |  |  |  |
| Price level of consumption |  |  | 0.005 |  |
|  |  |  | (0.018) |  |
| Public spending on agriculture |  |  |  | 0.015*** |
|  |  |  |  | (0.004) |
| Time effects | Yes | Yes | Yes | Yes |
| R-squared within | 0.706 | 0.355 | 0.625 | 0.488 |
| Number of observations | 300 | 171 | 557 | 287 |

Source: Authors’ estimates.

* *p* < 0.10. ** *p* < 0.05. *** *p* < 0.01. Standard errors are in parentheses. All explanatory variables are in logs.

**Table A5. List of countries in the sample**

Algeria Peru

Argentina Philippines

Australia Portugal

Austria Romania

Bangladesh Senegal

Benin South Africa

Brazil Spain

Bulgaria Syrian Arab Republic

Cameroon Thailand

China Tunisia

Costa Rica Turkey

Denmark Uruguay

Dominican Republic Zambia

Ecuador Zimbabwe

Finland

France

Gabon

Ghana

Greece

Hungary

India

Indonesia

Ireland

Italy

Jamaica

Japan

Jordan

Kenya

Malaysia

Mexico

Morocco

Nepal

Netherlands

New Zealand

Nicaragua

Panama

Paraguay

**Figure A1. LOWESS and scatter plots of the association between the percentage of 6-24 month old children having a minimal acceptable diet (MDD) and the diversity of food supply (share of calories from non-staples)**

Source: Authors’ estimates from FAO (2016) and DHS (2016) data.

Note: The solid line is an lpoly plot estimated in Stata 14. LOWESS = locally weighted scatterplot smoothing. Three-letter World Bank country codes denote specific observations. The full list of corresponding country codes can be found at: <http://wits.worldbank.org/WITS/wits/WITSHELP/Content/Codes/Country_Codes.htm>
